# Recent Developments in Chitosan-Based Micro/Nanofibers for Sustainable Food Packaging, Smart Textiles, Cosmeceuticals, and Biomedical Applications

**DOI:** 10.3390/molecules26092683

**Published:** 2021-05-03

**Authors:** Nguyen D. Tien, Ståle Petter Lyngstadaas, João F. Mano, Jonathan James Blaker, Håvard J. Haugen

**Affiliations:** 1 Department of Biomaterials, Institute of Clinical Dentistry, University of Oslo, 0317 Oslo, Norway; d.t.nguyen@odont.uio.no (N.D.T.); spl@odont.uio.no (S.P.L.); 2 CICECO–Aveiro Institute of Materials, Department of Chemistry, University of Aveiro, 3810-193 Aveiro, Portugal; jmano@ua.pt; 3 Department of Materials and Henry Royce Institute, The University of Manchester, Manchester M13 9PL, UK

**Keywords:** chitosan nanofibers, sustainable food packaging, advanced textiles, biofunctionalized materials, wound care, skin graft substitute, dermal regeneration

## Abstract

Chitosan has many useful intrinsic properties (e.g., non-toxicity, antibacterial properties, and biodegradability) and can be processed into high-surface-area nanofiber constructs for a broad range of sustainable research and commercial applications. These nanofibers can be further functionalized with bioactive agents. In the food industry, for example, edible films can be formed from chitosan-based composite fibers filled with nanoparticles, exhibiting excellent antioxidant and antimicrobial properties for a variety of products. Processing ‘pure’ chitosan into nanofibers can be challenging due to its cationic nature and high crystallinity; therefore, chitosan is often modified or blended with other materials to improve its processability and tailor its performance to specific needs. Chitosan can be blended with a variety of natural and synthetic polymers and processed into fibers while maintaining many of its intrinsic properties that are important for textile, cosmeceutical, and biomedical applications. The abundance of amine groups in the chemical structure of chitosan allows for facile modification (e.g., into soluble derivatives) and the binding of negatively charged domains. In particular, high-surface-area chitosan nanofibers are effective in binding negatively charged biomolecules. Recent developments of chitosan-based nanofibers with biological activities for various applications in biomedical, food packaging, and textiles are discussed herein.

## 1. Introduction

### 1.1. Preparation and Properties of Chitosan

**Figure 1:**
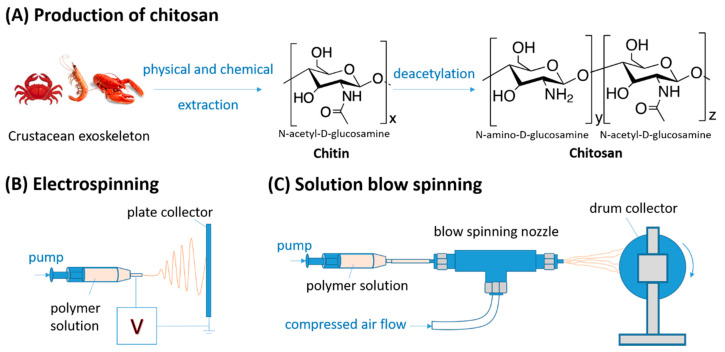
(**A**) The production of chitosan: Chitosan is made by deacetylation of chitin extracted from crustacean exoskeleton. Chitosan nanofibers are commonly fabricated by (**B**) electrospinning or (**C**) solution blow spinning, usually from chitosan dissolved in acetic acid. The principle of electrospinning is the induction of a liquid jet from a syringe nozzle using a high voltage, while solution blow spinning uses a high-pressure inert gas as a driving force. The solvent evaporates at the nozzle, and the polymer chains are stretched and travel the tip-to-collector distance, where the solidified nanofibers accumulate on the collectors.

Chitosan is presently one of the most attractive, sustainable biopolymers in use due to its availability and remarkable intrinsic properties, such as its digestibility, bacteriostatic and anti-inflammatory effects, biocompatibility, and biodegradability [1,2,3]. Chitosan is derived from chitin, which is extracted from crustacean shells (e.g., from crabs, lobsters, shrimps, or prawns) and mushrooms [4] through several processing methods involving demineralization and deproteination. The chitin is then converted to chitosan through a partial or full deacetylation process [5]. The production route and chemical structure of chitosan are described in Figure 1A.

Chitosan has a rigid D-glucosamine repeat unit, which consists of two monomers: β-(1-4)-2-acetamino-2-deoxy-β-d-glucose (*N*-acetyl-d-glucosamine) and β-(1-4)-2-deoxy-β-d-glucopyranose (*N*-amino-d-glucosamine) [6]. There are three functional groups in each repeating unit of chitosan: primary hydroxyl, secondary hydroxyl, and amine groups. Chemical modifications commonly target the amino groups to obtain desired properties and distinctive biological functions [7,8]. The degree of deacetylation (DDA) of chitosan is generally defined as the *N*-amino-d-glucosamine/*N*-acetyl-d-glucosamine ratio, an important indicator that determines the distinction between chitin and chitosan, thus leading to the noteworthy properties of chitosan. When the DDA is higher than 50%, the polymer is commonly called chitosan, becomes soluble in aqueous acidic media and is considered a cationic biopolymer due to protonation of the amino groups [9]. In terms of biological activity, a high DDA results in better compatibility and increases the interaction between chitosan and cells [10,11,12]. However, a low DDA induces an increase in the secretion of osteoprotegerin and sclerostin (SOST) relative to a high DDA [13]. Moreover, the combination of a high DDA and a high molecular weight (*M*_w_) has been shown to increase secretion of vascular endothelial growth factor (VEGF) and interleukin 6 (IL-6) but reduce secretion of osteopontin (OPN) compared to chitosan with a similar DDA but a lower *M*_w_. Manipulation of DDA and *M*_w_ therefore offers a strategy to tailor chitosan to specific industrial or biomedical requirements [14,15]. However, acetyl groups are removed during the deacetylation process, causing changes in *M*_w_ that must be taken into consideration when designing chitosan-based materials. Similarly, the *M*_w_ of chitosan (commonly ranging from 300 to 1 million) can affect its antibacterial properties [16,17], by which it impairs bacterial physiological activities at the cellular level [18]. Molecules with lower *M*_w_ degrade faster than those with higher *M*_w_, and molecules with low *M*_w_ and low DDA are more reactive as substrates but more vulnerable to biological degradation and chemical decay [19].

### 1.2. Fabrication of Chitosan-Based Micro/Nanofibers

Among the different physical forms of chitosan (e.g., powder, film, foam, and gel), chitosan-based nanofibers have drawn much attention due to their unique characteristics, such as their large surface-area-to-volume ratio and good mechanical strength when blended with other polymers [17]. Like most natural polymers, chitosan-based nanofibers can be made to retain biodegradable and biocompatible properties, which are of interest for applications in areas such as agriculture, packaging, or biomedicine [20].

Due to its versatility, electrospinning currently remains the most commonly used technique for the production of submicron-sized fibers since its emergence in the 1970s [21,22]. The principle of this technique is the formation of a liquid jet induced by an electrical field (Figure 1B). After solvent evaporation, a solid fiber is formed and deposited on the collector. However, electrospinning has a number of drawbacks. First, it requires hazardous operating conditions, such as high voltages and the use of toxic volatile organic solvents [23,24,25,26]. Furthermore, the effects of environmental conditions (ambient temperature and humidity) and other process variables make reproducibility a serious challenge [27]. The low deposition rates, which are typically on the order of 1 mL/h for lab spinning, result in low fiber production yield [28,29]. These factors all significantly challenge the commercial applicability of this method and its potential for the industrial scale-up of chitosan fiber production. Regardless of these drawbacks, various designs of the electrospinning method have been developed for mass production of nanofibers with satisfactory morphological accuracy. These designs are based on either multi-needle electrospinning or centrifuge electrospinning to increase fiber homogeneity, yield, and reproducibility, while needle-less electrospinning is used in an effoew5rt to reach industrial production yields [30,31,32].

A more recent and disruptive technique for nanofiber fabrication is solution blow spinning (SBS) [33]. A schematic drawing is shown in Figure 1C. This method offers high throughput and fiber diameter ranges similar to those of electrospinning without the need for high voltage and can be advantageous for ensuring high production yields. The SBS technique has been applied in both wet spinning (e.g., collection in coagulation baths) and dry spinning modes. In both of these modes, typically a polymer solution passes through the inner nozzle of a spinneret and a high-pressure gas (e.g., air, nitrogen, or argon) is emitted from the outer nozzle(s). Solvent from the solution jet is forced to evaporate, and the polymer chains are stretched and the tip-to-collector distance is projected, resulting in a solid fiber reaching the collector. A liquid collector bath is used for wet spinning; it is also possible to apply an electric field, and this technique is termed electro-blow spinning [34].

### 1.3. Modified Chitosan Micro/Nanofibers and Their Applications

It is challenging to fabricate chitosan as a submicron-sized fibrous form because chitosan has rigid D-glucosamine repeat units and the propensity to form inter- or intramolecular hydrogen bonds, leading to poor solubility in pure water as well as other common organic solvents. It has been shown that lowering the pH increases the water solubility of chitosan due to the protonation of primary amines [6]. The electrostatic repulsive forces between positive ammonium groups prevent intrachain hydrogen bonding, while the formation of interchain hydrogen bonds with water molecules enhances chitosan solubility in aqueous acidic solutions. Acetic acid has remained the most commonly used to adjust pH. A high concentration of acetic acid in water has been used successfully as a solvent for the electrospinning of pure chitosan [35,36]. However, while lowering pH with acids decreases surface tension, it also has a contradictory influence on chitosan spinnability because it increases the viscosity of chitosan solutions [35]. Chemical modification has been used as another approach to increase the solubility and spinnability of chitosan. Examples of chemically modified chitosan derivatives are hexanoyl chitosan [37], PEGylated chitosan [38], carboxyethyl chitosan [39], and quaternized chitosan [39,40]. As a result, chitosan derivatives are not only soluble in acidic aqueous solutions but also in neutral and basic aqueous solutions [3,41]. For example, introducing carboxymethyl to the chitosan structure can greatly enhance the water solubility of chitosan [42]. However, the most facile method of improving the spinnability of chitosan involves blending chitosan with another polymer of either natural or synthetic origin. Co-spinning agents have been widely investigated by research groups worldwide and included collagen [43], gelatin [44], cellulose [45], poly(ethylene oxide) (PEO) [46,47], poly(vinyl alcohol) (PVA) [48,49], poly(ε-caprolactone) (PCL) [50,51], and poly(lactide-*co*-glycolide) (PLGA) [52,53]. This strategy has produced electrospun chitosan/polyurethane nanofiber membranes with diameters ranging from 200 to 400 nm that can effectively filter microparticles larger than 475 nm [54]. However, efficient production of electrospun nanofiber membranes that can capture nanoparticles (smaller than 100 nm) remains difficult because the produced nanofibrous mats typically exhibit large variations in inter-fiber pore sizes and distributions. The membrane’s filter functions can be enhanced by altering the fibers’ electrostatic charges to promote chemical/physical interactions between them and between the fibers and the captured particles. It has been suggested that by utilizing the positive charge and excellent spinning properties of chitosan derivatives, a substrate for electrostatic viral repulsion (or capture) could be designed for personal protective clothing against the coronavirus SARS-CoV-2 as well as other viruses [55,56].

The use of micro/nanofibers in the design and development of innovative products is a rapidly rising topic in the field of materials research. Aiming to highlight the future importance of natural polymers and their potential use in smart materials, this review focuses on the recent development of micro/nanofibers based on chitosan as well as its derivatives, blends, and composites. Challenges, trends, and opportunities in the use of chitosan-based nanofibers for food, textile, cosmeceutical, and biomedical applications are discussed herein.

## 2. Chitosan-Based Nanofibers in Food Storage

The use of chitosan for food packaging in the form of films and edible coatings has been well documented [57,58,59]. Chitosan films, while sustainable and environmentally friendly, typically have poor mechanical and barrier properties. Moreover, edible coatings and packaging that require direct contact with food may influence its taste. This problem is particularly salient for materials based on fibers composed of chitosan derivatives due to the solvents and chemicals that are presently used in fiber spinning [57,58].

Nanofiber technology can potentially fabricate ultra-thin non-woven mats for food packaging applications [60]. Nanofibers with high chitosan content are used for antibacterial packaging, preserving the quality and safety of food products throughout distribution and storage due to their physical and chemical properties [61,62,63,64]. The antioxidant effect of chitosan is useful as a functional ingredient to improve the shelf lives of delicate food products [65]. From an environmental standpoint, packaging materials composed of chitosan nanofibers are bio-friendly, made from sustainable sources, and inherently biodegradable. Common methods of acquiring chitosan-based nanofibers for the food industry include functional components, also known as bioactives; these methods produce bio-functionalized nanofibers with specific properties.

**Figure 2:**
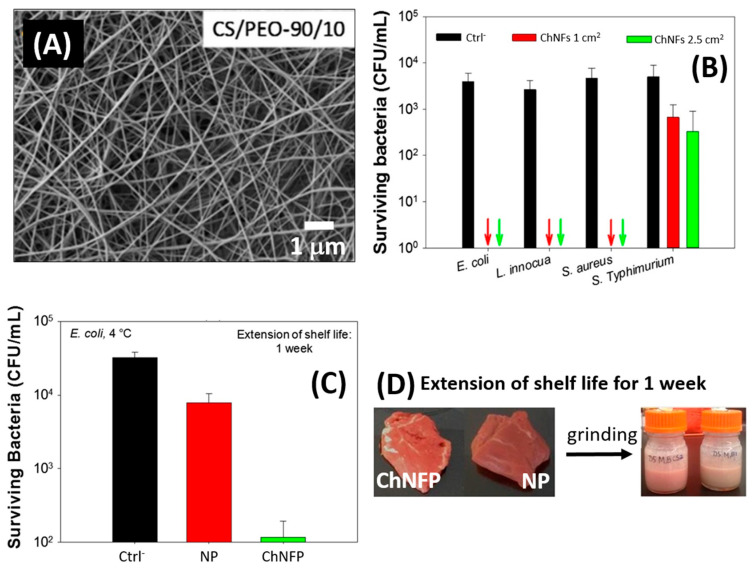
(**A**) An SEM picture of electrospun chitosan-PEO nanofibers (ChNFs) obtained from a 90/10 (*w/w*) solution in 50% acetic acid. (**B**) In vitro antibacterial activity of 1-cm^2^ and 2.5-cm^2^ swatches of ChNFs against *E. coli*, *L. innocua*, *S. aureus*, and *S.* *Typhimurium* compared to a negative control membrane (Ctrl^−^). The arrows indicate total inhibition of *E. coli*, *L. innocua*, and *S. aureus* growth. The wider the area of ChNFs (i.e., larger chitosan content), the better the effect against *S.* *Typhimurium.* (**C**) In situ antibacterial activity of ChNFs-based packaging (ChNFP) against *E. coli* compared to negative control (Ctrl^−^) of inoculated meat and positive control of inoculated meat wrapped in neat conventional packaging (NP) after seven days of raw meat storage at 4 °C. (**D**) Appearance of red meat wrapped in ChNFP and NP one week before and after grinding. Reprinted with permission from [66], copyright 2018 Wiley.

Chitosan/PEO nanofibers (ChNFs) have been developed as the inner part of multilayer packaging that prolongs the quality of unprocessed red meat [66]. The preparation of this material involves spinning the nanofibers directly on top of the multilayer packaging material to form ChNFs-based packaging (ChNFP). ChNFs containing 90 wt% chitosan electrospun from 50% acetic acid solution have been reported to produce a smooth and homogenous nanofiber layer (Figure 2A) [66]. In a test of bacteria that are commonly responsible for food alteration and poisoning, the prepared ChNFs were found to stop the growth of *E. coli*, *L. innocua*, and *S. aureus* while slowing the propagation of *S.* *Typhimurium* in 2.5-cm^2^ nanofiber mats compared to a negative control sample (Figure 2B). Under representative storage conditions in a refrigerator, inoculated *E. coli* bacteria were reduced by 95% after seven days of storage in fresh meat wrapped in contact with ChNFP compared to meat wrapped with conventional packaging (Figure 2C); therefore, ChNFP reduced meat spoilage and maintained the appearance and smell of raw meat (Figure 2D).

The encapsulation of bioactive components into chitosan fibers is another way to utilize chitosan composite nanofibers in food packaging. Glucose oxidase (also termed herein notatin) is a promising bioactive that produces hydrogen peroxide and D-glucono-5-lakton in the presence of sugar and oxygen. Notatin has been integrated into chitosan/PVA/tea extract nanofibers to prevent food spoilage through antimicrobial activity and deoxidation [67]. The obtained membrane inhibited the growth of microbes under low oxygen conditions (up to 73% deoxidation). Recently, natural food preservatives have been applied to chitosan nanofibers. For example, pomegranate peel extract (20 mg/mL) has been encapsulated into a ChNFs mat for the fabrication of a food wrapping film to enhance the shelf life of bovine meat without losing its fresh smell [68]. Similarly, an antibacterial assay of ChNFs-loaded tea oil liposomes inhibited 99.99% of *Salmonella sp.* in chicken meat, effectively keeping the meat fresh after four days of storage [69]. In another study, the encapsulation of liquid smoke/thymol into chitosan nanofibers significantly delayed the microbiological spoilage of fresh fish fillets [70].

It is worth underlining that chitosan-based nanofibers are emerging as an effective platform for enzyme immobilization (like notatin in the example above) in food processing due to their surface properties, which enhance enzyme activity [65,66,67]. Furthermore, chitosan fibers can also be functionalized with enzymes for a controlled release of additives or catalytic products. Additionally, liquid smoke/thymol can be added to chitosan nanofibers to ensure long-term release properties [70]. A recent study showed that electrospun chitosan/xanthan gum nanofibers can be used as delivery carriers for curcumin to increase its physical stability in aqueous media [71]. The future use of chitosan nanofibers for integration into food packaging materials and food processing could potentially improve the efficacy, sustainability, and storage capacity of food production and holds promise for more environmentally friendly food distribution.

## 3. Chitosan-Based Microfibers Applied in the Textile Industry

Chitosan is used extensively in textile dyeing and finishing due to its polycationic nature, which allows it to be combined with anionic dyes or to form strong ionic bonds with fabric materials [69,70]. However, there is far less literature on chitosan nanofibers than on synthetic polymer fibers in the textile industry. One often mentioned application of the nanofibrous structure of chitosan is the development of wound dressing materials. These dressings can carry and deliver pharmaceutical compounds when in contact with wounds and ulcers. Wound dressings are highlighted as one of the most attractive applications of electrospun chitosan nanofibers for medical textiles [72]. Similarly, metallic nanoparticles can be incorporated into chitosan fibers for use in other medical textiles. These textiles are designed for diagnostic or therapeutic purposes, such as biosensors and drug carriers. Biomedical applications are discussed in detail separately in Section 5. For non-biomedical textiles, there is little available documentation on the use of chitosan nanofibers, even though their potential uses are many. Herein, we focus on the recent developments of chitosan-based microfibers for the fabrication of smart textiles (also termed herein biofunctional textiles) [73].

**Figure 3:**
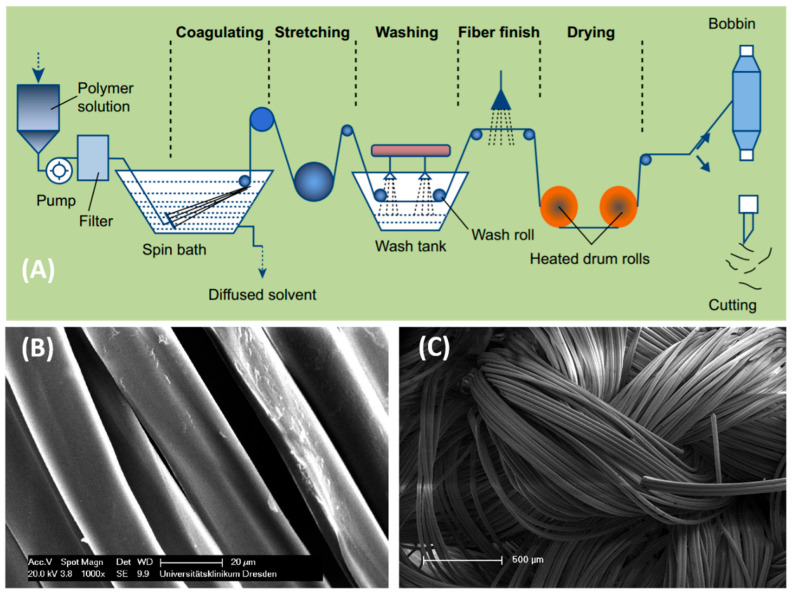
(**A**) Schematic illustration of the wet spinning process for yarn preparation adopted from [74]. (**B**) Chitosan microfibers wet spun from an 8.5 wt% solution. (**C**) The constructed knitted fabric. Reprinted with permission from [75], copyright 2014 Taylor & Francis.

A straightforward method of preparing pure chitosan microfiber yarns is wet spinning, though these approaches normally result in fibers >5 µm in diameter. Some perspectives on wet-spun chitosan and chitosan-blends are given in this section. There is an opportunity to use some newer disruptive spinning approaches such as solution blow spinning, electrospinning, and electro-blow spinning with coagulation baths to render these submicron fibers in processing. The typical processing stages for traditional wet-spinning of chitosan fibers are illustrated in Figure 3A [74]. Chitosan microfibers can be wet spun from a viscous chitosan solution (up to 8.5 wt%) in acetic acid [75]. The obtained microfibers have an average diameter of approximately 20 µm (Figure 3B), resulting in remarkable tensile strength (28.7 N, Young’s modulus; 12.2 GPa) and elongation at break (3.8%). The knitted fabrics were also constructed with 76–600 multi-filaments with strong breaking tenacity (Figure 3C). Using a similar technique, the obtained chitosan-grafted polyethylene glycol monomethyl ether (chitosan-*g*-mPEG) fiber improved by more than 50% in tensile strength and 200% in tensile modulus in contrast with the pure chitosan fiber [76]. These improvements were due to the enhancement of molecular entanglement and hydrogen bonding interactions. Moreover, the chitosan-*g*-mPEG fiber could regulate temperature through solid–solid phase change behavior. Therefore, this fiber would be a good choice for the fabrication of thermo-responsive fibers for smart textiles. Chitosan fibers with higher strength (by up to four times) were obtained by applying a glycine chloride ionic liquid spinning solution instead of the acetic acid solution [77]. Furthermore, the addition of inorganic nanoparticles (e.g., silver or gold) into chitosan-spinning dope could create functionalized knitted fabrics for use in textiles, dressings, scaffolds, and surgery meshes [78].

The production costs of pure chitosan fibers and finished textile products are high because they require many modification steps compared to commonly used synthetic polymers. Therefore, researchers typically blend chitosan with other polymers to reduce production costs and obtain good mechanical properties while maintaining the positive properties of chitosan. Interestingly, the use of natural polymers as alternatives for synthetic polymers (e.g., cellulose/chitosan composite fibers spun from a mixture of polymers in an aqueous solution of LiOH/KOH/urea) has produced microfibers with excellent mechanical properties in both dry and wet states [79]. This observation gives hope for the sustainable textile industry for less reliance on synthetic polymers.

**Figure 4:**
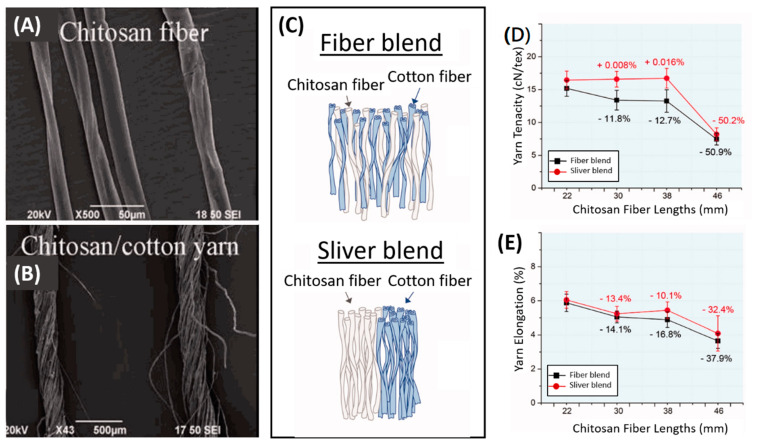
Micrograph images of surface structures in (**A**) chitosan fiber and (**B**) chitosan/cotton yarn. (**C**) Illustration of the blending methods. The yarn fiber blend showed higher (**D**) tenacity and (**E**) elongation than the yarn sliver blend at all chitosan fiber lengths. Reprinted with permission from [80,81], copyright 2014 & 2017 SAGE Publications.

A commercial blending approach known as fiber blending or yarn blending is favored because it can be used to manufacture large-scale productions in traditional spinning mills. Chitosan-cotton-blended yarns were produced using this method, and the effects of different blending methods (fiber blending and sliver blending) on the tensile properties of the yarn were studied (Figure 4C) [80]. The chitosan fibers exhibited grooves on their longitudinal surfaces (Figure 4A) that could enhance friction between chitosan and chitosan-cotton fibers during blending, thus increasing the electrostatic charge and resulting in hairy structures (Figure 4B). With respect to mechanical properties, cotton fiber blending produced better tenacity and elongation properties than sliver blending (Figure 4D,E). Considering the chitosan fiber lengths produced by both blending methods, a decrease in tensile properties was observed along with an increase in fiber lengths. Given that the positive charges of chitosan molecules limit their textile applications due to their high electrostatic behavior, differences in yarn performance between chitosan/cotton-blended yarns and chitosan/polyacrylonitrile-(PAN)-blended yarns were studied by the same group [81]. The researchers selected two polymer fibers with opposite electric charge properties via friction—negative PAN fibers and positive cotton fibers—to blend with positive chitosan fibers. The tenacity of the chitosan/PAN-blended yarns was found to be superior to that of the chitosan/cotton-blended yarns. Given that the total charge approach was neutral when PAN was blended with chitosan, this mix was preferred for maximizing yarn performance and minimizing wastage.

All in all, advanced materials using mixes of natural polymers for smart and sustainable textiles have emerged as good candidates for the replacement of environmentally harmful synthetics and could significantly lower carbon dioxide emissions [82]. Aside from cellulose, chitin and chitosan are under consideration by the textile industry due to their sustainability and green credentials [83,84].

## 4. Chitosan-Based Nanofibers Applied in Cosmeceuticals

Due to the recent development of nanomaterials and biotechnology, chitosan and its derivatives are among the natural materials of interest for use in cosmeceuticals. These materials can act as either oral hygiene agents or nanocarriers for active compounds in cosmetic and personal care products [85,86]. The intrinsic antioxidant activity of chitosan is beneficial for skincare products due to its potential anti-aging effects [87]. In particular, the antioxidative carboxylation of chitosan inhibits the activity of matrix metalloproteinases (MMPs) that dissolves the collagen matrix of connective tissues and protects against reactive oxygen species (ROS) that harm tissues during radiation treatment, sunburns, and biological stress [88]. The strong antibacterial activity of chitosan against *Streptococcus mutans*, a major cause of dental decay, has made way for the addition of chitosan nano/microparticles to oral healthcare products, such as toothpastes and mouth rinses [89,90]. Finally, chitosan and its derivatives can also interact with keratin, forming elastic and resistant films on individual hair strands that protect against wear and damage, thus enhancing the appearance and conditioning of hair [91].

**Figure 5:**
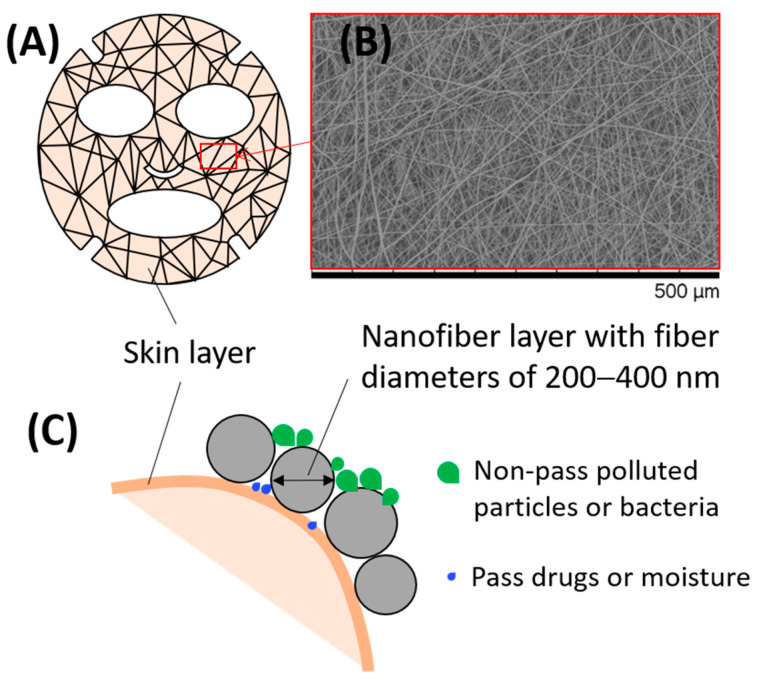
(**A**) A protective cosmeceutical mask for facial skincare after burns based on (**B**) a chitosan nanofiber membrane. (**C**) Schematic illustration of the nanofiber layer on the skin surface. Depending on the materials and active ingredients used in formulation for manufacturing, the nanofiber face mask can exhibit various functionalities, including increased contact area with the skin, protection of the skin from microsized polluted particles and bacteria, and maintenance of skin conditioning and moisture.

Although chitosan derivatives have been applied as innovative materials for the manufacture of various cosmeceutical products, only a limited number of cosmeceutical products utilize chitosan nanofibers. To the authors’ knowledge, the only cosmeceutical product reported to utilize these nanofibers is a chitin nanofibril mask designed for use as a facial dressing for medical purposes; the nano/micropores in this product allow bioactive substances through the dressing and block microbes and contaminants from reaching the healing skin [92]. This mask has been described as a transparent, flexible, and thin film made by casting technology. Nanofibrous chitosan and its derivatives can be used to cast custom-made dressing scaffolds with antioxidant and antimicrobial properties. Skin-friendly dressing filters based on these nanofiber membranes typically contain preservatives, drugs, and/or active healing agents and can be prepared by either electrospinning or blow spinning, producing a functionalized nanofiber layer for wound healing, skin therapy, and other cosmeceutical applications (Figure 5).

## 5. Chitosan-Based Nanofibers in Biomedical Applications

To date, the most important achievements in the application of chitosan-based nanofibers have been in the biomedical field. This is due to the unique hemostatic properties of these nanofibers, which promote platelet aggregation and neutrophil/macrophage activities [93]. For example, chitosan nanofibrous scaffolds can offer an excellent biocompatible environment for cell seeding and the promotion of tissue growth. One of the major challenges of tissue engineering is that of finding appropriate biodegradable materials that are non-toxic and compatible (physiologically and biochemically) with the human body. As mentioned previously, chitosan can be blended with natural and/or synthetic polymers to improve the spinnability, stability, mechanical properties, and biofunctionality of nanofibers and scaffolds. An abundance of bioactives, drugs, and therapeutic compounds have been incorporated into chitosan nanofibrous meshes to meet specific requirements as wound healing agents, antimicrobial agents, growth factors, and drug carrier agents [94]. This section reviews specific examples of chitosan nanofiber-based materials for wound healing, targeted delivery of bioactive compounds, and tissue engineering.

### 5.1. Wound Healing

**Figure 6:**
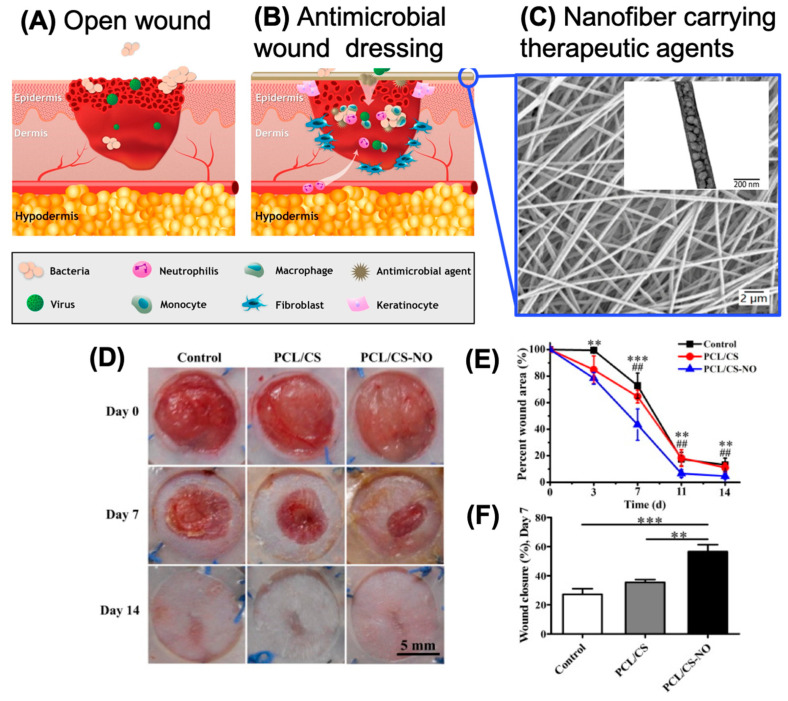
The wound healing process of (**A**) an open wound covered with (**B**) an antimicrobial wound dressing. When the antimicrobial dressing covers the wound bed, it acts as a barrier to prevent bacteria and viruses from invading the wound. Dressings with embedded functional agents can promote the healing rate and stimulate the immune system to speed up skin regeneration in immunocompromised patients. Reprinted with permissions from [101], copyright 2018 Elsevier. An example in (**C**) shows chitosan-based nanofibers with 0.3% *w/v* of polyhexanide (a commonly used wound disinfectant) embedded, used to fabricate an antimicrobial surface on a surgical dressing. Reprinted with permissions from [102], copyright 2020 Springer. (**D**) In vivo wound healing in a mouse model using a PCL-chitosan nanofibrous dressing with or without incorporated nitric oxide. The nitric oxide-containing material promotes faster wound closure, as illustrated in (**E**) by the percentage of open wound area and in (**F**) by the percentage of wound closure. Reprinted with permissions from [109], copyright 2018 Elsevier. ** *p* < 0.01, *** *p* < 0.001, ## *p* < 0.05.

Wound healing is a dynamic process consisting of four phases: hemostasis, inflammation, growth, and remodeling through a timed sequence [95]. Protective patches (i.e., wound dressings) have probably been used to treat wounds while they heal for as long as humans have lived, but wound dressings still have a long way to go before they can improve and/or speed up the natural healing process. There is still a grave need for multifunctional wound dressings that can aid in the healing of advanced chronic ulcers in immunocompromised patients [96]. Various designs of wound dressings in the form of films, hydrogels, sponges, and foams made of natural or synthetic materials (or combinations of these materials) are currently in use [97]. Wound dressings made from chitosan-based nanofibers have emerged as a new class of wound dressings that show superior properties in terms of biocompatibility, biodegradability, porosity, and antimicrobial activity. These dressings can also provide an optimum moisture environment, absorb wound exudate, and (in some cases) accelerate wound recovery [98]. Aside from chitosan, the pros and cons of a variety of biopolymers used in wound treatments have been extensively covered by literature reviews [96,99,100]. The schematic illustration in Figure 6A,B shows the application of a polymeric wound dressing with antibacterial activity to cover an open wound [101]. Such a functional nanofiber-based dressing not only protects the wound but can also modulate the release of therapeutic agents embedded in the fibrous network to aid in healing (Figure 6C) [102].

In one study, researchers reported that composite nanofibers made from honey, tripolyphosphate, and chitosan loaded with capsaicin and gold nanoparticles can enhance the wound closure rate through antibacterial effects and increased dermal cell proliferation in the nanofibrous scaffold [103]. In another study, blended chitosan nanofibers (also called hybrid fibers) prepared by electrospinning were used to manufacture a bicomponent nanofibrous mat containing chitosan and poly(lactide) (PLA) [104]. This mat effectively inhibited the growth of *S. aureus* and *E. coli* bacteria in wounds compared to a corresponding solvent-cast chitosan-PLA film. It has also been shown that core-sheath-structured PLA-chitosan nanofibers can promote fibroblast adhesion and proliferation [105]. Aside from PLA, PEO and PCL are also frequently used to fabricate hybrid fibers together with chitosan due to their interaction with tissue and favorable mechanical properties [50,106]. Natural ingredients, such as aloe vera, have also been used in chitosan-PCL nanofibers to improve the hydrophilicity and antibacterial properties of the hybrid material [107]. More structurally advanced, electrospun multilayer scaffolds have also been fabricated for wound healing [108]. A first layer providing mechanical support is composed of PCL or a PCL/cellulose acetate blend, while a second layer designed to come in direct contact with the wound surface is composed of ChNFs. These scaffolds exhibited a porosity of 85% with high water vapor permeation, making them suitable for treating an exudative wound. These scaffolds also allow for swelling up to 370% without any loss of mechanical or structural properties and are biocompatible with fibroblast cells, making them potentially useful as resorbable dressings for full-thickness wounds.

### 5.2. Delivery of Bioactive Compounds

Bioactive molecules and therapeutic compounds have been combined with chitosan nanofiber membranes to further improve their performance in wound dressings. One approach to improving the performance of chitosan-based wound dressings involves incorporating inorganic nanoparticles into the chitosan-containing nanofibers to promote wound healing and/or improve their antibacterial potential. Nitric oxide (NO), which plays a vital role in the wound healing processes from inflammation to tissue remodeling, has been adsorbed into electrospun mats of PCL/chitosan nanofibers by drop coating [109]. The NO released from these materials helps to accelerate the closure of acute, experimental wounds (Figure 6D–F). Other inorganic compounds that have wound healing properties and have been incorporated into chitosan nanofibers include ZnO [110], Fe_3_O_4_ [44], TiO_2_ [111], and various metals (gold, silver, and copper) [112]. It should be noted that, depending on the context and application, silver and copper are not only antimicrobial but can be toxic and thus should be applied with caution [113,114]. It is also worth mentioning that the incorporation of metals into nanofiber scaffolds can provide electrical conductivity to the fibers and help generate electric or magnetic fields with various applications, such as wearable smart textiles, biosensors, or the direction of cell migration. For example, loading magnetic nanoparticles (Fe_3_O_4_) into chitosan nanofibers allows the materials to be heated, making them effective for hyperthermia treatment [115].

Another composite natural nanofiber system has been reported to contain fibers of 40 wt% N-carboxyethyl chitosan (a water-soluble chitosan derivative) and 60 wt% PVA, with incorporated silk fibroin nanoparticles. These fibers were fabricated by electrospinning [116]. The presence of silk fibroin nanoparticles lent more strength to the material and facilitated increased fibroblast cell adhesion and growth but caused the material to degrade faster in biological systems.

Chitosan-based nanofibrous materials for use in wound care can be further improved by adding multiple biochemical stimuli, e.g., growth factors and antibacterial molecules. In one study, the researchers loaded two synergetic growth factors into ChNFs [117]. When applied, these fibers release a burst of vascular endothelial growth factor (VEGF), a key mediator for angiogenesis and granulation tissue formation, which accelerates early-stage healing, followed by a sustained release of platelet-derived growth factor-BB (PDGF-BB), which promotes connective tissue growth and remodeling.

For the treatment of severe burns, 2% (*w/v*) bromelain (a debriding agent commonly used in burn treatment) was added to electrospun chitosan nanofibers [118]. When applied to burns, this material reduced the burn wound area and significantly increased the density of collagen tissue compared to ordinary passive burn dressings.

Similarly, *Zataria multiflora* (ZM) essential oil is a strong natural antimicrobial agent and has been incorporated into electrospun chitosan/PVA/gelatin nanofibers to fabricate wound dressings for use after surgery and on burn wounds [119]. These nanofibers were loaded with 10% (*v/v*) ZM and completely stopped the growth of bacteria over 24 h of incubation. Although this material was highly effective against microbial growth, its strength was significantly reduced. Several other natural healing agents have also been tested as adjuvants to composite chitosan nanofibers with varying results, including a curcumin-loaded chitosan/poly(propylene carbonate) [120] and a henna-leaf-extract-loaded chitosan-based scaffold [121], both of which showed positive effects on acute wound healing in experimental models.

Finally, the combination of different chitosan derivatives can also be used to fabricate advanced active wound dressings. In one experiment, electrospun chitosan/PVA nanofibers were manufactured with different concentrations of carboxymethyl-chitosan-encapsulated antibacterial peptide nanoparticles (OH-CATH30, a clinical drug) [49]. This material showed dual antibacterial activity and promoted acute wound healing. The resulting nanofiber system was not only suitable for use as a wound dressing but was also recognized as a good drug-carrier platform for other biomedical applications.

### 5.3. Tissue Engineering

#### 5.3.1. Skin

Wound dressings based on nanofibers can be effective in protecting wounds from contamination and infections and can be modified to promote skin repair and therefore shorten healing time. These devices may also be designed to deliver therapeutic drugs [122]. Most dressings are thin isotropic materials that can restrict the migration of cells through thickness. Acting as a barrier to growth and migration, this can cause skin cells to behave abnormally and cause aberrant skin healing with fibrosis and scarring [123]. This shortcoming is particularly salient for deep, full-thickness wounds involving both the epidermis and the dermis [124]. It has been suggested that using nanofibers to construct a 3D scaffold that mimics the structure of the skin’s extracellular matrix could produce better conditions for cell growth and promote skin tissue repair and regeneration [125]. Such novel materials could also have a multi-layered design with zones that mimic the layers of the skin to provide a multidimensional roadmap for cells to complete the healing of full-thickness wounds.

Several authors have recently reported on experiments combining nanofibers with hydrogels in a 3D structure for tissue engineering and wound care [126,127,128]. One experiment investigated a bilayer scaffold consisting of hybrid nanofibers made of human hair keratin and chitosan for epidermal cell ingrowth and a gelatin-methacrylate hydrogel to support the connective tissue cells of the dermis [128]. The use of chitosan nanofibers together with keratin could potentially overcome the poor mechanical properties of chitosan while enhancing the structural stability of the keratin substrate. This artificial skin structure has shown some promise in the lab but lacks in vivo data; thus, it is difficult to evaluate this structure’s potential for clinical use.

**Figure 7:**
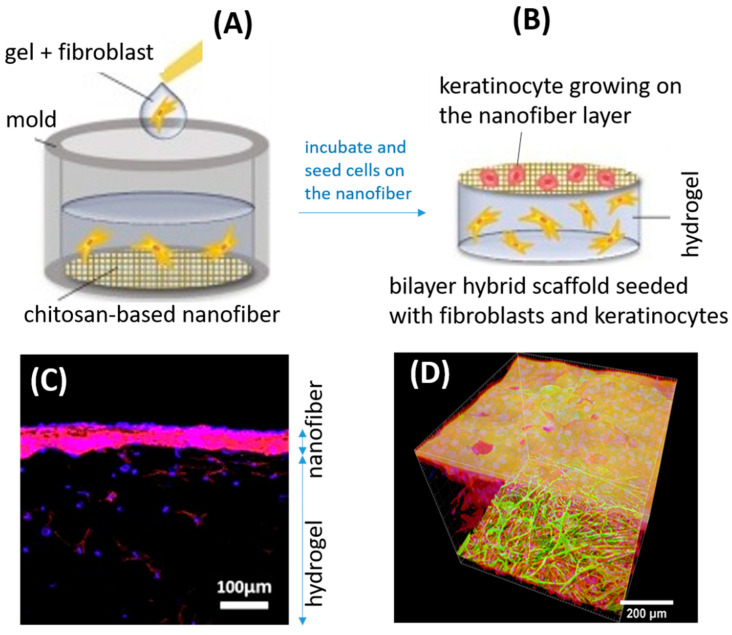
A bilayer scaffold composed of a nanofiber mat and a hydrogel mimics the layered nature of skin. The chitosan-based nanofiber was prepared by electrospinning, while the hydrogel was synthesized by photopolymerization. (**A**) Human fibroblasts were encapsulated in the gel and poured into a mold with the nanofiber mat placed on the bottom. (**B**) After a short incubation period, the hydrogel was flipped over with the nanofiber mat side up, and keratinocyte cells were seeded onto the nanofiber surface. After co-culturing, encapsulated fibroblasts (blue) proliferated in the hydrogel matrix, while keratinocyte cells (red) formed a layer on top of the fibrous scaffold, mimicking the epidermis and the dermis. (**C**) A cross-section of the cellularized bilayered material. Reprinted with permissions from [128], copyright 2017 Elsevier. (**D**) A 3D scaffold made from a nanofiber-hydrogel composite and captured by confocal microscopy. The fibrin-coated nanofiber mat (green) enabled the fibroblasts (red) to migrate from the nanofibers and into the collagen hydrogel (middle layer), mimicking the skin’s dermis. Different from the 3D scaffold in (**B**), the hydrogel in (**D**) constitutes the top layer supporting the embedded keratinocytes (yellowish), mimicking the skin’s epidermis. Reprinted with permissions from [129], copyright 2019 Dove Medical Press.

In co-culture tests with this two-layered structure, fibroblasts were added to the hydrogel part and keratinocyte cells were added to the nanofiber mesh on the opposite side (Figure 7A,B). Cell populations then migrated and proliferated, forming distinctively different layers: an epidermis-like layer on the nanofiber mesh and a dermis-like layer in the hydrogel (Figure 7C). In a similar but contrary approach, a two-layer skin construct consisting of an epidermal side made of collagen hydrogel reinforced by a fibrin-coated polylactide nanofibrous membrane on the epidermal side was manufactured and tested in co-cultures of dermal cells [129]. The dermal layer was a fibrin-coated poly(L-lactide) nanofibrous membrane that integrated fibroblasts, mimicking the dermis layer of the skin. A collagen-based hydrogel was then added on top of the fibrous membrane and seeded with keratinocytes, which spread out to form a homogeneous basal layer that mimicked the epidermis layer of the skin (Figure 7D). Chitosan nanofibers were not used in this material, but it is envisaged that the added value of chitosan could make this construct more bioactive, stronger, and more protective against microbial invasion when applied in the clinic.

In general, skin is an extremely hyperelastic material with the capacity to reorganize its structure, to repeatedly stretch far beyond its normal dimensions and return to its original state when strain is released, without any loss of mechanical integrity. An optimal skin-mimicking material must therefore be sufficiently tough and elastic to last long enough for the skin to heal. Significantly, the skin-mimicking strategies described in the present literature are lacking in mechanical properties such as tension, torsion, and viscoelasticity. In addition, key information on the clinical performance of these strategies is lacking, especially with respect to vascularization, cell differentiation, tissue integration, degradability, and infection control. This may hinder our understanding of the mechanism of vascularization for tissue-engineered skin products. More work is needed to consolidate the proof of concept for complex, biomimicking skin substitutes. However, it seems reasonable that the versatility, availability, and favorable biological, environmental, and mechanical properties of chitosan nanofibers make them natural constituents of future skin tissue engineering materials.

#### 5.3.2. Bone

Chitosan is structurally similar to glycosaminoglycan, which is found in bone; thus, chitosan is considered to be one of the most promising natural biopolymers for bone tissue engineering [130,131]. A bone biomimetic scaffold based on nanocomposite nanofibers of chitosan and hydroxy-apatite (HAp; 70/30 *w/w*) was synthesized by electrospinning [132]. The obtained nanofibers had an average diameter of approximately 214 nm and contained proper, structurally preserved, HAp nanocrystallites. When tested in an experimental model for bone growth, the nanofiber scaffold showed promising osteoconductive properties. In a further development, collagen (7.2 wt%) was added to the electrospun chitosan/HAp fibers in an effort to enhance osteoblast-scaffold interaction and promote bone formation. Experiments demonstrated that this chitosan/HAp/collagen biocomposite scaffold significantly improved bone formation compared to HAp alone [133]. In another recent study, a scaffold of electrospun ChNFs reinforced with halloysite nanotubes (aluminosilicate clay mineral, HAL) was designed and fabricated for bone regeneration [134,135,136,137]. These nanofibers incorporated approximately 5 wt% HAL and had a diameter of approximately 120 nm. The resulting scaffold material exhibited a porous structure with high tensile strength. When tested in experimental models, the material showed promising osteoinductive properties that make it a good candidate for testing as a bone graft substitute material.

## 6. Future Prospects

Recently, significant progress has been made in the fabrication of multicomponent, multifunctional chitosan-based nanofiber systems [54,102]. Functionalizing these fibers with various bioactives, drugs, therapeutic agents, and conductive or magnetic components is now a common strategy to further boost and tune the properties of chitosan and its derivatives [49,94,138]. However, while these technologies are currently available, challenges remain since adding materials could be sensitive to the processing environment (heat, humidity, or pH), potentially losing functionalities in applications. Therefore, chitosan-based fibers still require further development and testing for improved function both as medical devices and commercial products in daily life.

The connection between the biological properties and physical characteristics of these fibrous materials remains to be investigated and fully understood on all scales. For example, advanced nanofiber configurations (e.g., core-sheath nanofibers, aligned nanofibers, and gradient nanofibers) show different biological and mechanical properties [139]. This implies that controlling the physical shapes and chemical configurations of these fibers is instrumental to the design of smart materials with superior nanoscale properties, such as controlled-release functions, the guiding of cell adhesion and migration, conductivity and magnetism for biosensor applications, improved strength and elasticity, and potentially nanoscale self-assembly and self-disassembly (e.g., for injectable biomedical remedies and perpetual sustainable recirculation) [140,141,142]. Further post-spinning treatments could also improve adhesion between fibers for improved fabrics in food packaging or smart clothing or for improved bioactivity in biomedical applications. Each surface modification strategy has its potential in the development of multifunctional fibrous scaffolds. By combining process parameters of pre- and post-spinning treatments, researchers can create advanced scaffolds with tuning properties while preserving nanofiber integrity.

Moreover, the completely degradable nature of chitosan-based materials requires further attention. Given that the degradation profile is directly related to the precise chemical structure of the polymer and the hierarchical architecture of the fibers, the degradation of chitosan-based fibrous materials can be tailored [143,144]. This property alone, especially if it can be controlled and triggered, holds great promise for sustainable food, cosmeceutical, and biomedical applications.

A major future application of chitosan-based nanofibers is in tissue engineering. Materials scientists are designing more and more advanced materials for wound care and tissue graft substitutes. Although fiber spinning is evidently a versatile technique for sub-micron fiber production, it still has limitations, for example the reproducibility due to a large number of controlled parameters and subsequently affecting dimensions and morphology of final product requirements [145,146,147]. Due to increased regulatory restrictions particularly in Europe (European Tissue and Cells Directive [148]) and the new medical device regulation (MDR) [149], on fibers from animal sources, we are likely to see a shift in use of collagen fiber for medical use towards more sustainable biomaterials such as chitosan [150]. The combination of advanced fabrication methods (e.g., sub-micron fiber spinning and 3D printing methods) would help to produce multidimensional chitosan-based materials with structures that mimic some aspects of natural tissues. These materials have shown outstanding biological qualities while also exhibiting viscoelastic properties, high compressive strength, and potential for controlled release and resorption [151]. Together, these properties make chitosan-based materials ideal as a basis for developing the next generation of smart materials for tissue engineering purposes.

A selection of commercial chitosan-based materials based on non-woven fibers that are available for advanced wound care market is summarized in Table 1. Although chitosan-based micro/nanofibers have successfully been scaled-up and entered several clinical trials, issues regarding mass production of sub-micron chitosan fibers are still unsolved. To ensure commercial success for sub-micron chitosan fibers in the medical market, challenges such as homogeneity of raw materials, reproducibility, regulatory obstacles, and production cost must be addressed.

## 7. Conclusions

Chitosan-based micro/nanofibers have demonstrated a desirable platform for a broad spectrum of applications. An overview of recent developments in chitosan-based micro/nanofibers has been summarized in this review (shown in Table 2). The desired intrinsic properties of chitosan conformed into high-surface-area nanofiber constructs can now be used as edible coatings to preserve the food quality, non-woven fabrics with sustainability and green credentials, antibacterial face masks in cosmetics, or biomedical substrate for skin and bone tissue regeneration. Owing to the latest advances in materials science in synergy with biotechnology, chitosan is seen as valuable natural raw material for novel products that facilitate healthy and sustainable living.

## Figures and Tables

**Table 1:** Commercial chitosan-based fiber products on the medical wound care market.

Material	Product Name	Specification	Approved Market	Manufacturer
Chitosan	Celox^TM^ Rapid	Hemostatic dressings contain activated chitosan bonded to a high-density gauze	USA, EU	Medtrade Products Ltd., Crewe, UK
Chitosan	axiotstat^®^	Hemostatic dressings have mucoadhesion due to charge	USA, EU	Axio Biosolutions Pvt. Ltd., Ahmedabad, India
Chitosan	ChitoFlex^®^ PRO	Hemostatic dressings for the treatment of moderate to severely bleeding wounds	USA, EU	Tricol Biomedical, Inc., Portland, Oregon, USA
Chitosan	ChitoSAM^TM^100	Hemostatic dressings optimized to stop the bleed fast	USA, EU	SAM^®^ Medical, Tualatin, Oregon, USA
Chitosan	ChitoClot Gauze	Hemostatic dressings reduce bleeding time	USA, EU, Taiwan	BenQ Materials Corporation, Taoyuan, Taiwan
Chitosan	Chitoskin^®^	A non-woven chitosan-based skin substitute	China	Hainan Xinlong Nonwovens Co., Ltd., Haikou, China
Chitosan	Chito-Seal	Hemostatic dressings for bleeding wounds	USA	Abbott Vascular, Inc., Chicago, Illinois, USA
Chitosan	Clo-Sur P.A.D^®^	A non-woven topical pad accelerates hemostasis	USA	Scion BioMedical, Miami, Florida, USA
Chitosan	ExcelArrest^®^ XT	Hemostatic dressings made of modified chitosan that accelerates the clotting process	USA, EU	Hemostasis, LLC, Saint Paul, Minnesota, USA
Chitosan/alginate	Tromboguard^®^	Act as the contact layer of a multilayer hemostatic dressing	EU	TRICOMED S.A., Łódź, Poland
Chitosan/rayon	ChiPro face mask	Chitosan face masks contain 45% chitosan and 55% rayon	Germany	ChiPro GmbH, Bremen, Germany
Chitosan/polynosic	Chitopoly^®^	Antimicrobial wears made of chitosan and polynosic fiber	Japan	Fuji Spinning Co., Ltd., Tokyo, Japan
Chitosan/viscose	Crabyon^®^	The Crabyon^®^ fiber that made of chitosan and viscose used for the textile market	Switzerland	Swicofil AG, Lucerne, Switzerland
Chitosan	Chitopack C^®^	Cotton-like chitosan prepared by wet spinning	Japan	Eisai Co., Ltd., Tokyo, Japan

**Table 2:** Summary of chitosan-based micro/nanofibers for sustainable food packaging, smart textiles, cosmetics, biomedical applications, and their characteristics.

Micro/Nanofibers	Composition	Fiber Diameter	Application	Remark	Reference
Chitosan/PEO	(10−90):(90−10) (*w/w*) 3:1, 1:1, 1:3 (*w/w*)	<500 nm	Food packaging Wound healing Bone tissue engineering	- Developed as the inner part of multilayer packaging that preserves the quality and freshness of meat - Can be fabricated core/shell nanofibers	[66,106,108,117,135,136]
Chitosan/PEO/HAL	1:1 (*w/w*):(1−10 wt%)	70−160 nm	Bone tissue engineering	High tensile strength with a porous structure and good biocompatibility	[134]
Chitosan/PEO/PE ^1^	(80−20):(20−80) (*w/w*): (20 mg/mL PE)	211−421 nm	Food packaging	Preserve and enhance the shelf life of beef	[68]
Chitosan/PEO/TTO ^2^	20:2 (mg/mL): (30−70% TTO) (*v/v*)	150−300 nm	Food packaging	Prevent the microbial contamination by *Salmonella* to extend the shelf life of chicken meat	[69]
Chitosan/PVA/tea extract/GO ^3^	(10−50):(90−50) (*w/w*):(1.5 wt% tea extract): (28−250 mg GO)	100−120 nm	Food packaging	Have bacteriostasis and deoxidizing ability that prolong the shelf life of food	[67]
Chitosan/PVA/ZnO (or Ag, Cu, *Zataria multiflora*)	1:4 (*w/w*):-	270−320 nm	Wound dressings Cosmetic	Have good antibacterial and antioxidant properties that served as dressings for diabetic wounds	[49,110,112,119]
Chitosan/PVA/silk fibroin	40:60 (*w/w*):(4, 8 wt%)	126−643 nm	Wound dressings	Good biocompatibility with mouse fibroblasts (L929)	[116]
Chitosan/thymol/liquid smoke	6.75% (*w/v*):0.4% (*v/v*):0.2% (*w/v*)	72−132 nm	Food packaging	Delay growth of mesophilic bacteria in fish fillets	[70]
Chitosan/xanthan gum/curcumin	3%:0.75%:2% (*w/v*)	750−910 nm	Functional packaging	- Stable nanofibrous structures in aqueous media - High encapsulation efficiency	[71]
Chitosan + gelatin	8.5 wt% (chitosan) 15−30 wt% (gelatin)	20 µm (chitosan) 130 nm (gelatin)	Functional textile	- Chitosan microfibers coated gelatin nanofibers- High mechanical strength with the cell-seeding ability	[75]
Chitosan/gelatin/Fe_3_O_4_ (or cinnamon extract)	1:1(*w/w*):(0.5−4 wt%)	307−435 nm	Wound dressings	Enhance mechanical and antibacterial properties	[44,137]
Chitosan/mPEG	1:(1−5) (*w/w*)	-	Smart textile	- High tensile strength - Able to regulate temperature	[76]
Chitosan/glycine chloride ionic liquid	6.5%:4% (*w/w*)	20 µm	Smart textile	Strong mechanical properties due to high orientation and crystallinity of fibers	[77]
Chitosan + cotton	50:50	-	Functional textile	- High tensile strength - High uniformity of fiber distribution	[80]
Chitosan + PAN	12:88, 30:70, 50:50	-	Functional textile	- High tenacity - Reduce electrostatic charges	[81]
Chitosan/nylon-6	3%:21% (*w/v*)	200−350 nm	Wound healing	Fabricated as core/shell antimicrobial nanofibers	[102]
Chitosan/PLA	50:50 (*w/w*)	840 nm	Wound healing	Suppress the adhesion of bacteria *S. aureus* and *E. coli*	[104,105]
Chitosan/PCL/aloe vera	(1−3):(8−5) (*w/w*):(1−3 wt%)	<100 nm	Wound dressings Cosmetic	High antibacterial performance and biocompatibility	
Chitosan/PPC ^4^/curcumin	1:2 (*w/w*):10 wt%	200−400 nm	Wound healing	Enhance wound healing efficacy	[120]
Chitosan/HAp	70:30 (*w/w*)	195−240 nm	Bone tissue engineering	Stimulate the bone forming ability	[132,133]
Chitosan/collagen/PEO/polypyrrole	2.6:0.6:2.1 (*w/v*): (5−25 wt%)	83−140 nm	Skin tissue engineering	Conductive nano-scaffolds with high biocompatibility	[138]

^1^ PE: pomegranate peel extract, ^2^ TTO: tea tree oil liposomes, ^3^ GO: glucose oxidase, ^4^ PPC: poly (propylene carbonate).
